# Differentially expressed genes in the femur cartilage transcriptome clarify the understanding of femoral head separation in chickens

**DOI:** 10.1038/s41598-021-97306-3

**Published:** 2021-09-09

**Authors:** Ludmila Mudri Hul, Adriana Mércia Guaratini Ibelli, Igor Ricardo Savoldi, Débora Ester Petry Marcelino, Lana Teixeira Fernandes, Jane Oliveira Peixoto, Maurício Egídio Cantão, Roberto Hiroshi Higa, Poliana Fernanda Giachetto, Luiz Lehmann Coutinho, Mônica Corrêa Ledur

**Affiliations:** 1 Programa de Pós-Graduação em Ciências Veterinárias, Universidade Estadual do Centro-Oeste, Guarapuava, PR 85040-080 Brazil; 2 Embrapa Suínos e Aves, Concórdia, SC 89715-899 Brazil; 3 Programa de Pós-Graduação em Zootecnia, Centro de Educação Superior do Oeste (CEO), Universidade do Estado de Santa Catarina, UDESC, Chapecó, SC 89815-630 Brazil; 4 Faculdade de Concórdia FACC, Concórdia, SC 89701-130 Brazil; 5 Embrapa Informática Agropecuária, Campinas, SP 70770-901 Brazil; 6 Departamento de Zootecnia, Escola Superior de Agricultura “Luiz de Queiroz”, Universidade de São Paulo, Piracicaba, SP 13418-900 Brazil

**Keywords:** Animal breeding, Functional genomics

## Abstract

Locomotor problems are among one of the main concerns in the current poultry industry, causing major economic losses and affecting animal welfare. The most common bone anomalies in the femur are dyschondroplasia, femoral head separation (FHS), and bacterial chondronecrosis with osteomyelitis (BCO), also known as femoral head necrosis (FHN). The present study aimed to identify differentially expressed (DE) genes in the articular cartilage (AC) of normal and FHS-affected broilers by RNA-Seq analysis. In the transcriptome analysis, 12,169 genes were expressed in the femur AC. Of those, 107 genes were DE (FDR < 0.05) between normal and affected chickens, of which 9 were downregulated and 98 were upregulated in the affected broilers. In the gene-set enrichment analysis using the DE genes, 79 biological processes (BP) were identified and were grouped into 12 superclusters. The main BP found were involved in the response to biotic stimulus, gas transport, cellular activation, carbohydrate-derived catabolism, multi-organism regulation, immune system, muscle contraction, multi-organism process, cytolysis, leukocytes and cell adhesion. In this study, the first transcriptome analysis of the broilers femur articular cartilage was performed, and a set of candidate genes (*AvBD1*, *AvBD2*, *ANK1*, *EPX*, *ADA*, *RHAG*) that could trigger changes in the broiler´s femoral growth plate was identified. Moreover, these results could be helpful to better understand FHN in chickens and possibly in humans.

## Introduction

In the last decades, an intense selection has been performed for greater feed efficiency and faster growth of broiler chickens^1^. However, a significant increase in locomotor problems has been detected, causing a negative impact on welfare, feed efficiency, performance and other characteristics^2,3^, and consequently, economic losses^2^. These losses are due to increase in mortality, reduction in feed conversion and weight gain caused directly or indirectly by skeletal problems^4^. Currently, bone disorders are still considered one of the main concerns for the poultry industry^5^. Among the locomotor problems, bacterial chondronecrosis with osteomyelitis (BCO) is the most common cause of claudication, affecting approximately 1.5% of chickens slaughtered at 42 days of age in the United States, as well as an important cause of mortality in broilers^6^. This condition, also known as femoral head necrosis (FHN)^7^, is one of the most important disturbances in the locomotor system in commercial chickens worldwide^8^. Besides its importance in poultry production, there are few studies on this pathology, especially related to its genetics and molecular mechanisms^5,7,9–12^.

The separation of the articular cartilage (AC) from the growth plate (GP), known as proximal femoral head separation (FHS) is a risk factor for infection that may cause BCO in broilers^13,14^. This can occur because the BCO pathogenesis seems to be initiated by damage of the poorly mineralized chondrocyte (cartilage cells) columns in the epiphyseal and physeal growth plates of the leg bones, followed by colonization of the osteochondral clefts by opportunistic bacteria^6,7^.

Genetics play a considerable role in the skeleton development, where genetic selection and gene mutations influence the development of the skeletal system^2^. DNA mutations in different genes are involved with different skeletal clinical phenotypes^15^. There is a controversy in the literature regarding BCO and FHN terminology. Some authors consider BCO and FHN as the same pathology^16,17^, while others consider them different pathologies^9,18,19^. Usually, when there is separation of the AC and necrosis, this condition is called FHN, while when there is evidence of bacterial infection, it is referred to as BCO^17^. Previous studies evaluating the chicken femoral growth plate found that the genes *RUNX2*, *SPARC*^11^, *ADIPOQ, PRRX1, ANGPTL5, ANGPTL7, GFRA2, SFRP5, COL14A1, ABI3BP, COL8A1, SLC30A10*^20^, *bFGF*^5^, *LEPR*^21^, *LRP1B*, *COL28A1*, *PTHrP*, *PERP1*, *FAM180A* and *CHST1*^12^ were associated with the FHN. According to the Chicken Quantitative Trait Loci (QTL) Database (Chicken QTLdb) (https://www.animalgenome.org/cgi-bin/QTLdb/GG/summary), some QTL for morphometric traits, mineral composition, and tibia and femur resistance were mapped to several chicken chromosomes, indicating important regions related to bone development^22^.

Recently, differences in the cartilage morphology and metabolism were observed when normal and FHN-affected broilers were compared (Liu et al. 2021). However, there are no studies investigating the molecular and genetic pathways involved with FHS in broilers articular cartilage, and its etiology is still unknown in chickens^22,23^. The functional analysis of genes is important to elucidate their contribution to the development of locomotor problems in chickens. Therefore, this study aimed to identify differentially expressed (DE) genes in the femoral head articular cartilage between healthy and FHS-affected broilers using RNA-Seq analysis.

## Results

### RNA sequencing and differential expression analysis

The sequencing of the femoral head articular cartilage samples generated around 190 million (2 × 100 bp) reads. An average of 26.82 million paired-end reads was obtained per sample, remaining about 23.7 million after the QC. Approximately, 94.6% of the reads were mapped against the chicken reference genome (GRCg6a) available at Ensembl 95. The percentage of reads mapped per sample ranged from 93.45 to 95.2% and were similar between the normal and FHS-affected group (Supplementary File 1: Table S1). The MDS plot showed the separation between the affected and control samples evaluated in the current study (Supplementary File 2: Fig S1).

A total of 12,169 genes were expressed in the femoral articular cartilage, of which 107 genes were differentially expressed (FDR < 0.05) between the two groups (Supplementary File 1: Table S2). From those, 91 were annotated and 16 were uncharacterized (Table 1). Out of the 107 DE genes, 98 (91.6%) were upregulated and 9 (8.4%) were downregulated in the FHS-affected compared with the healthy control group (Supplementary File 1: Table S2). Hierarchical clustering analysis of the 107 DE genes showed different expression patterns between FHS-affected and control sample groups and homogeneity among samples from each group (Supplementary File 2: Fig S2).

**Table 1:** Characterization of the articular cartilage transcriptome showing the total number of expressed genes and the differentially expressed ones, according to Ensembl 95 gene type information.

Gene type	Expressed genes	DE
lncRNA	409	5
miRNA	18	0
IG_V_gene	3	0
Pseudogenes	154	2
snRNA	10	0
rRNA	2	1
Misc_RNA	1	0
Mt_rRNA	2	0
Protein-coding genes	10,496	83
Uncharacterized-protein genes	1,074	16
Total	12,169	107

Considering the top 9 DE genes, those related to collagen (*COL13A1*), myosin (*MYH15*), phosphatases (*PSPH*) and transferases (*GGT1)* were downregulated, while genes involved with response to microorganisms and immune system were upregulated in the FHS-affected group (Table 2).

**Table 2:** Top 9 downregulated and upregulated differentially expressed genes in the articular cartilage between normal and FHS-affected broilers.

Ensembl Gene ID	Gene symbol	Gene description	Log2FC
ENSGALG00000039489	*RF00002*	5.8S ribosomal RNA	− 2.23
ENSGALG00000010490	*DPYSL4*	dihydropyrimidinase like 4	− 2.00
ENSGALG00000015358	*MYH15*	Gallus gallus myosin, heavy chain 15	− 1.92
ENSGALG00000006565	*GGT1*	gamma-glutamyltransferase 1	− 1.50
ENSGALG00000038225	*SEMA3E*	semaphorin 3E	− 1.44
ENSGALG00000002397	*PSPH*	phosphoserine phosphatase	− 1.42
ENSGALG00000004286	*COL13A1*	collagen type XIII alpha 1 chain	− 1.29
ENSGALG00000043671			− 1.23
ENSGALG00000014686	*FBN2*	fibrillin 2	− 1.08
ENSGALG00000016669	*AvBD2*	Gallus gallus avian beta-defensin 2	3.66
ENSGALG00000019696	*CATHL2*	Gallus gallus cathelicidin antimicrobial peptide	3.69
ENSGALG00000028273	*HBE1*	Gallus gallus hemoglobin subunit epsilon 1	3.73
ENSGALG00000024272	*S100A9*	Gallus gallus S100 calcium binding protein A9	4.04
ENSGALG00000006572	*TNNT3*	troponin T3	4.04
ENSGALG00000023953	*C4BPA*	Gallus gallus complement component 4 binding protein	4.05
ENSGALG00000002907	*MYL1*	myosin, light chain 1	4.17
ENSGALG00000043254	*EPX*	eosinophil peroxidase	4.26
ENSGALG00000014463	*ACTN2*	Gallus gallus actinin alpha 2	4.33

### qPCR validation

In the qPCR analysis, six out of the 11 analyzed genes were DE between the FHS-affected and the normal group (Table 3). Moreover, the same expression profile between both approaches was observed for all evaluated genes, except for *FBN2* (Table 3), confirming the RNA-Seq results obtained in the current study.

**Table 3:** Relative expression (Log2FC) and statistical significance between normal and FHS-affected broilers obtained with qPCR and comparison with RNA-Seq results for 11 candidate genes.

Gene	qPCR	*p*-value	RNA-Seq	FDR
*AvBD1*	3.21	0.005	3.55	1.23E-07
*AvBD2*	3.65	0.006	3.32	0.0006
*FBN2*	0.52	0.227	− 1.08	0.037
*ANK1*	2.45	0.014	2.12	0.023
*RHAG*	3.04	0.006	2.44	0.010
*CSF3R*	0.86	0.356	2.28	0.003
*EPX*	3.48	0.007	4.27	3.23E-09
*ADA*	2.14	0.003	1.72	0.008
*COL13A1*	− 0.22	0.562	− 1.30	0.038
*S100A9*	2.51	0.093	4.04	1.84E-06
*PSPH*	− 1.13	0.218	− 1.43	0.012

### Gene ontology and gene network analyses

**Figure 1:**
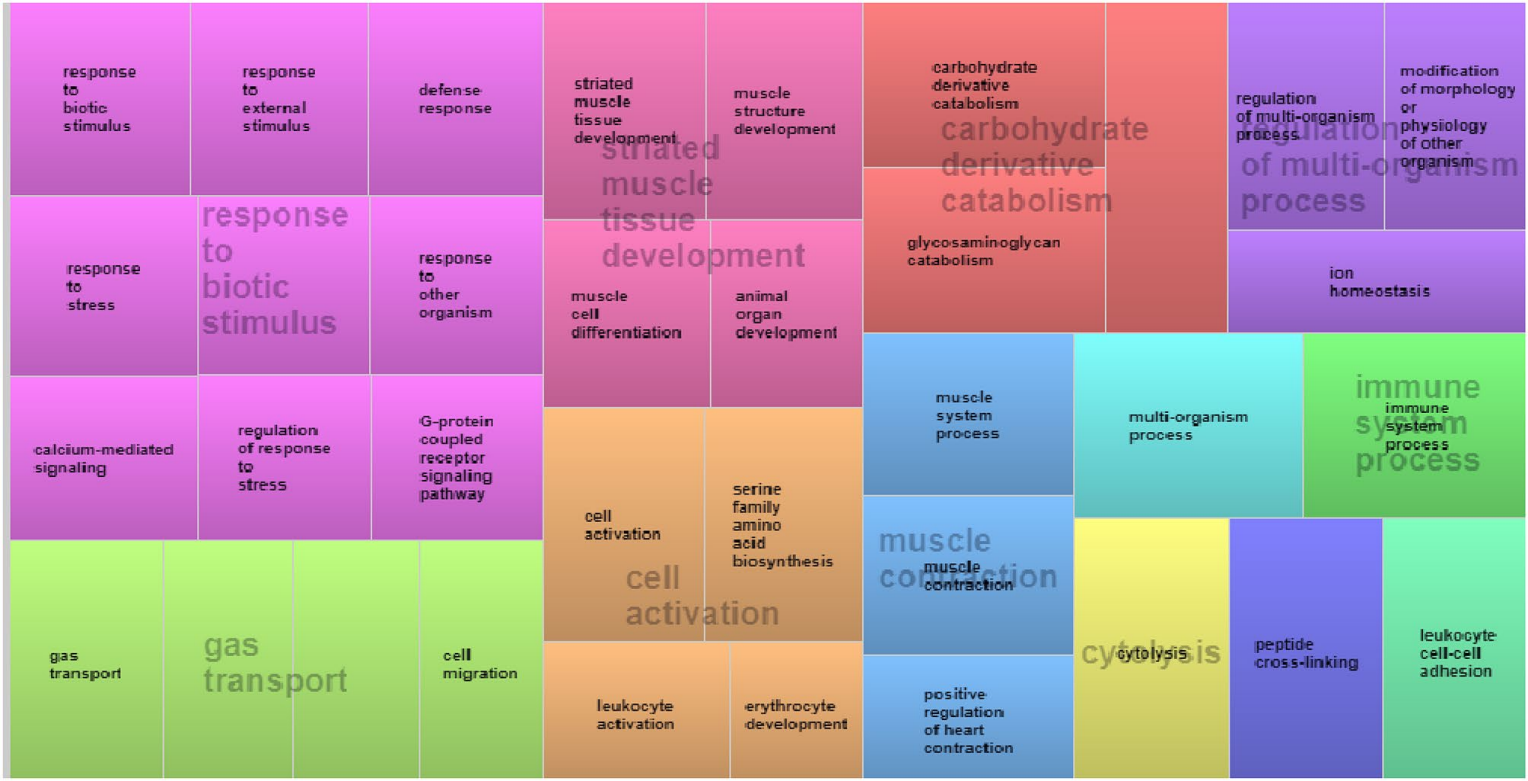
Superclusters of biological processes enriched for up- and downregulated genes in the articular cartilage related to FHS using REVIGO^104^. Different colors show different superclusters, and the size of each box is determined by the uniqueness of the categories.

In the gene ontology (GO) analysis, DE genes with defined biological functions were grouped into 79 functional clusters according to their most relevant biological processes (BP) identified through the GO enrichment analysis (Supplementary File 1: Table S3). These BP were summarized in 12 superclusters (Fig. 1). The main BP identified were related to immune system processes, response to biotic stimulus, striated muscle tissue development, carbohydrate derivative catabolism, gas transport and cell activation.

**Figure 2:**
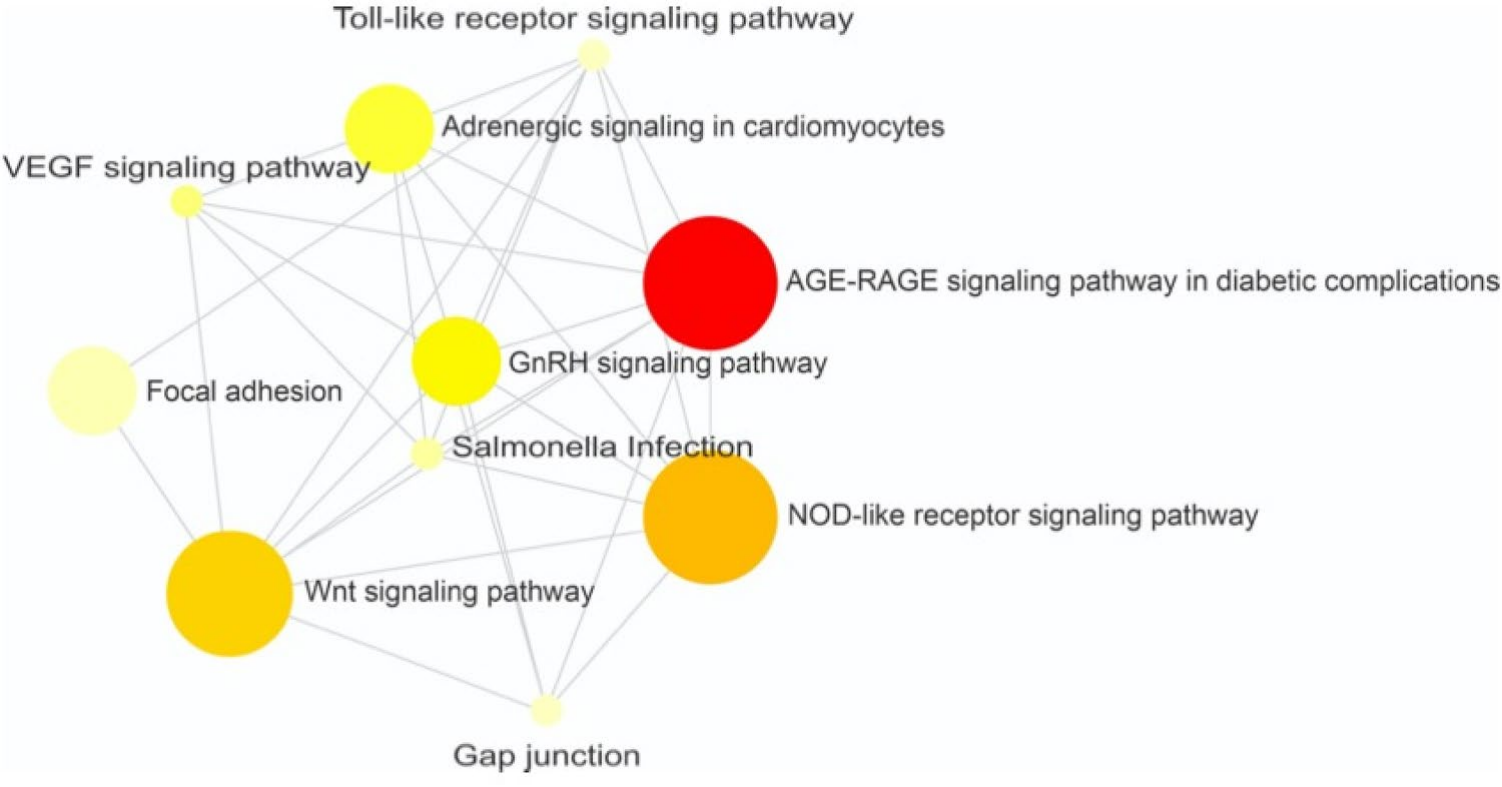
Gene network of differentially expressed genes-enrichment analysis using KEGG with chicken information. Circles represent metabolic pathways and connecting lines represent interactions between them, according to the active NetworkAnalyst prediction method. Nodes are colored according to their over-representation analysis (ORA) p-value. Darker colors are the smallest *p*-values. The node size is related to the number of genes enriched in each pathway.

**Figure 3:**
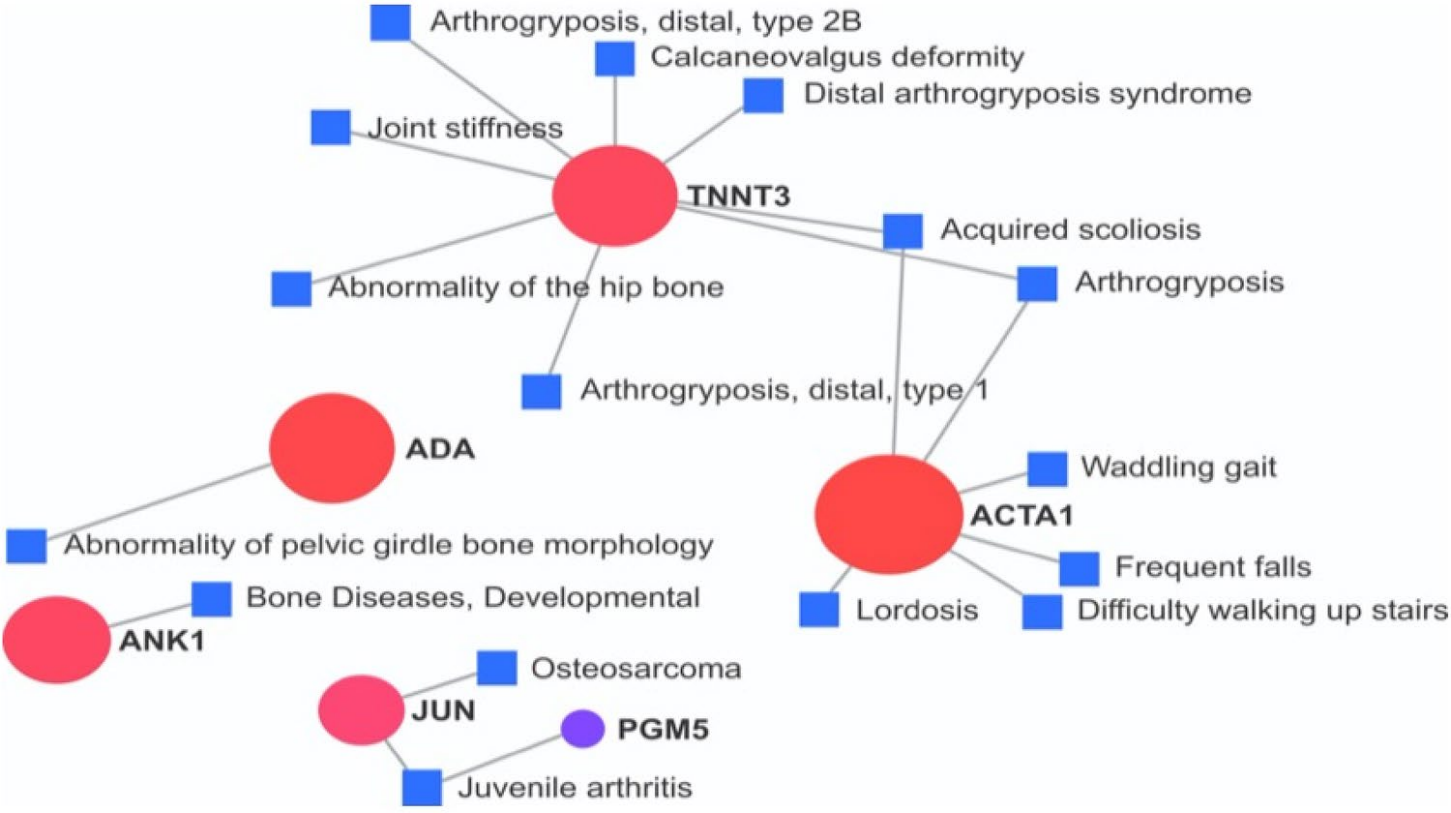
Gene network of differentially expressed genes constructed with the human gene-disease associations database (DisGeNET). In the figure, the genes previously associated with locomotor diseases in humans are shown. Circles represent the DE genes, the squares represent the diseases and connecting lines represent the association between the genes and bone/locomotor diseases, according to the active prediction method of NetworkAnalyst. The size of the circles was related to all conditions associated to the gene, however, for better visualization, we have shown only those related to bone/locomotor diseases.

To verify the known interactions among the DE genes aiming to improve the knowledge about them, an analysis at the NetworkAnalyst platform was performed with the chicken database. An enriched network was generated, where genes were linked to pathways, such as Wnt signaling pathway (*PLCB2, CCND3, JUN, RAC2*), GnRH signaling pathway (*PLCB2, MAPK12, JUN),* adrenergic signaling in cardiomyocytes (*PLCB2, MYH15, MAPK12*)*,* VEGF signaling pathway (*MAPK12, RAC2*)*,* bacterial infection (*MAPK12, JUN*)*,* focal adhesion (*CCND3, JUN, RAC2*), gap junction (*TUBB1, PLCB2*) and toll-like receptor signaling pathway (*MAPK12, JUN*) (Fig. 2, Supplementary File 1: Table S4). Furthermore, some DE genes were associated with bone-related disease problems when compared with a human database (Fig. 3). The *JUN* and *PGM5* genes were related to osteosarcoma, *ACTA1* with waddling gait and difficulty to walk, *ADA* with abnormality of pelvic girdle bone morphology, *TNNT3* with joint stiffness, arthrogryposis distal type 1, ulnar deviation of the wrist, ulnar deviation of the fingers, abnormality of the hip bone and *metatarsus varus*, and *ANK1* with bone development (Fig. 3).

## Discussion

Studies related to bone integrity problems, such as FHS and FHN or BCO are scarce in chicken models^7,11,12,24,25^. Most of the bone tissue studies reported to date are in humans and rodents^22,26–28^. Furthermore, there are no studies evaluating the transcriptional profile of femoral articular cartilage from chickens and its impacts in bone-related problems. Therefore, in the present study, a global gene expression profile of the femoral head articular cartilage of normal and FHS-affected broilers at 35 days of age was reported for the first time.

In the cartilage transcriptome, approximately 91.5% of the DE genes were upregulated while 8.5% were downregulated in the FHS-affected compared to the normal group (Supplementary File 1: Table S2). The expression profile of the AC tissue differed from the femoral growth plate reported in a previous study, in which approximately 49% of the genes were upregulated and 51% downregulated in the FHS-affected group^12^. In the femoral growth plate, several biological processes identified were related to angiogenesis, blood coagulation, cell adhesion, bone development and lipid metabolism, with several genes downregulated in the FHS-affected group^12^. Here, in the AC tissue, 79 BP were found (Supplementary File 1: Table S3) and, after REVIGO clusterization, the genes that draw the most attention due to their function were those related to response to biotic stimulus, immune response, cytolysis, striated muscle development, carbohydrate derivative catabolism and cell activation processes. Several BP were similar between the growth plate^12^ and the AC. However, in the AC transcriptome, a higher proportion of the genes had an increased level of expression in the affected animals than in the GP transcriptome. In our study, 107 genes were identified as DE in the AC and 28 of those were also found by Peixoto et al.^12^ in the bone GP. Moreover, some genes previously described as potentially involved with FHS, such as interferon alfa-6 inducible protein (*IFI6*)*,* adenosine deaminase (*ADA*)*,* cathelicidin-3 (*CATH3*)*,* avian beta defensin 1 (*AvBD1*)*,* avian beta defensin 2 (*AvBD2*)*,* ankirine 1 (*ANK 1*)*,* leukocyte cell-derived chemotaxin 2 (*LECT2*) and collagen type XII alpha chain 1 (*COL13A1*)^5,11,12,14,20^ were also DE in the current study*.* Among the DE genes, some of them (*AvBD1, AvBD2, ANK1, RHAG, ADA* and *EPX)* were confirmed by qPCR, which demonstrates the reliability of the results found with the RNA-Seq approach (Table 3).

Here, only 9 DE genes were downregulated in the cartilage of broilers with FHS, in which the *COL13A1* can be highlighted. This gene shows a wide tissue distribution and occurs at the cellular junctions and cell–matrix interaction sites in epithelial, mesenchymal, and neural tissues. It is a component of focal adhesion in cultured fibroblasts^29^ interacting with the collagen-binding integrin α1β1, suggesting its involvement in multiple cell–matrix interactions^30,31^. Therefore, the downregulation of *COL13A1* and possibly other collagen genes could reduce the synthesis of the extracellular matrix, facilitating the separation of the cartilage and bone tissues, favoring the occurrence of proximal FHS. Considering all downregulated DE genes, the BP involved were mainly related to muscle development processes (muscle cell differentiation, contraction, development, striated muscle cell development and tissue development) (Supplementary File 1: Table S3) and could be associated to the cytoskeleton, since the lower expression of these genes can lead to malformation in the femur cartilage structure, which could contribute to the separation of the articular cartilage from the growth plate.

Through the evaluation of the upregulated genes, most of the BP was involved with the recruitment of immune cells to enhance the adaptive immune response, blood circulation, angiogenesis, circulatory system development and cellular adhesion. The *CCND3, CDK6, JUN, ADD2, ANK1, RHAG, EPB42, SLC4A1, STOM, CAMP, SERPINB10, LYG2, CATHL3, AvBD1, AvBD2, S100A9, CSF3R, RAC2, FGL2, PTPRC, LYVE1* and *ITGAB2* genes (Supplementary File 1: Table S3, Fig. 1) can be highlighted due to their involvement in direct antimicrobial activities and immunomodulatory responses^32,33^. It is important to highlight that, since the information regarding FHN is scarce in chickens, it is hard to prospect BP and pathways involved with this condition. Moreover, as we had a relatively small number of DE genes, the non-corrected p-values were used for enrichment analysis. The main BP identified in the AC from DE genes between FHS-affected and normal broilers are discussed below.

### Genes related to immune response

Several studies have reported bacterial infection as one of the causes of the BCO. However, there is a controversy in the literature regarding the bacterial infections in the FHS and whether it is related or not to the cause of this condition^9,16–19^. The genes *MAPK12* and *JUN* were related to the bacterial infection and to the toll-like receptor signaling pathway BP. The later BP plays a key role in the innate immune system. The upregulation of those genes can be a consequence of the FHS, through the recognition of structurally conserved molecules derived from microbes that breached physical barriers, and are recognized by the toll-like receptors, activating the immune response^34^.

Immune biological processes had 18 genes present in DAVID and REVIGO (Supplementary File 1: Table S3, Fig. 1). The gene expression profile observed in this study showed a global activation of the immune system (Fig. 1). Among the enriched genes, *ANK1, AvBD1, ACTN2, ADA, C7, CATH2, CCND3, CSF3R, EDN2, JUN, TF, RHAG, S100A9, SERPINB10, SSTR2, AvBD2, EPB42, LECT2, LYG2, PTPRC* and *STOM* were upregulated in the FHS-affected group. The *AvBD1, AvBD2, AvBD7, CATHL2, CATHB1, LECT2, SERPINB10 and S100A9* genes were enriched in the host immune response BP. Mainly *AvBD1, AvBD2* and *CATHL2* are key components of the innate immune system^35,36^. *LECT2 (Leukocyte cell-derived chemotaxin 2)* encodes a multifunctional protein characteristically similar to cytokines that improve protective immunity in bacterial sepsis^37^.

The BP of defense response to other organisms was enriched with *RSFR, LYG2, AvBD1, STOM, CATH2, AvBD2* and *SERPINB10* genes. The identification of this BP indicates a probable presence of pathogenic microorganisms in the AC tissue. It has been suggested that BCO can be initiated by a mechanical micro fracturing of the growth plate, followed by colonization of osteochondrotic clefts by different opportunistic bacteria circulating in the blood^25^. Moreover, one of the causes of BCO is bacterial translocation from the intestinal tract and their proliferation in bone fissures^6^, and *Staphylococcus aureus* was found to be the most frequent bacteria associated to osteomyelitis^38,39^.

The host-defense peptides (HDPs) are a group of small molecules that have direct antimicrobial activities and immunomodulatory properties that are responsible for the recruitment of immune cells to enhance the adaptive immune response^32,33^. Its antimicrobial activity aims to eliminate bacteria, enveloped viruses, fungi and protozoa by binding the cell and producing pores that lead to cell leakage and lysis, while the immunomodulatory properties help boosting the adaptive immunity through chemotaxis of lymphocytes^40^. In avian species, three classes of HDP are described: avian beta-defensins (AvBDs), cathelicidins (CATHs), and liver-expressed antimicrobial peptide 2 (LEAP-2)^41,42^.

The AvBDs group comprises 14 genes, which encode proteins that are different in their chemical structure, mainly amino acid sequence and composition^43^. In this study, the beta-defensins *AvBD1* and *AvBD2* were upregulated in the FHS-affected broilers, confirming the possible role of these genes in controlling the FHS development and, in consequence, interrupting the FHS progression towards BCO.

The *ANK1* gene encodes a protein related to the binding of the structural constituent of the cytoskeleton, protein that aids in the attachment of other proteins in the membrane to the actin-spectrin cytoskeleton^44,45^. According to Hall et al.^46^, *ANK1* has an adaptive function as a membrane adapter protein, making connections between the cell membrane proteins and the spectrin-actin cytoskeleton, resulting in cell migration. Ankirin-1 has a role in supporting cell movement after damage. As the ankirin-1 can affect the structure of the actin filament and the cellular motility, it is possible that increased levels of ankirin-1 may inhibit the organization of the actin filament by increasing the binding of the spectrin-actin or, alternatively, the ankyrin-1 could act modulating the signaling pathways of actin remodeling^46^. Moreover, the gene *ANK1* is co-regulated by p53, which is involved in a variety of cellular functions, including cell-cycle arrest, DNA repair, and apoptosis^46,47^. The upregulation of *ANK1* can be related to the FHS and BCO since its high expression can alter the structure of actin cytoskeleton affecting the structural integrity of the femur articular cartilage, contributing to the occurrence of proximal FHS. On the other hand, *ANK1* expression is related to cellular damage, so it could also be a consequence, since after the damage process from the FHS is initiated, the upregulation of *ANK1* can act as a sign of trying to combat the progression of this condition.

The inflammation is a vital component of the host defenses, but on the other hand, excessive inflammation can cause tissue damage^48^. The adenosine deaminase *(*ADA*)* is an enzyme that acts as an endogenous regulator of the adaptive immune response, playing an important role on T-lymphocytes proliferation and differentiation^49^. Furthermore, adenosine regulates cell metabolism and triggers a variety of physiological effects in cell proliferation^50^. The *ADA* gene acts as a sensor and provides information to the immune system about tissue damage, protecting the host cells from excessive tissue injury associated with strong inflammation^51^. The upregulation of *ADA* could downregulate the activation of lymphocytes during inflammation, and also play a regulatory role on neutrophils in immune responses^50,52^. Extracellular adenosine signaling has been shown to play a role in inflammation during hypoxia and ischemia–reperfusion injuries, usually resulting in vascular leakage, accumulation of inflammatory cells, and elevated cytokine levels in serum. Moreover, just as hypoxia can induce inflammation, inflamed tissues often become severely hypoxic^53^. The *ADA* upregulation can be considered a consequence of the proximal FHS, since its high expression is related to immune responses, trying to combat inflammatory process already installed. Therefore, it gives rise to the hypothesis that through the increased number of bacteria in the tissue, there is an upregulation of the *ADA* gene, aiming to fight and eliminate the bacteria that are causing damage.

The *EPX* gene is activated during an immune response, releasing proteins and other components in the area of injury or inflammation that have a toxic effect on severely damaged cells or infecting pathogens. One of these proteins is called eosinophil peroxidase, that are extremely cytotoxic to bacteria^54,55^, parasites^56,57^, eukaryotic cells^58^ and neoplastic cells^59,60^. The upregulation of the *EPX* may be a consequence of FHS, since a possible infection could pressure the bone structure, impairing the blood supply to the affected area, developing necrosis. Furthermore, *EPX* could regulate the inflammatory process to control the infection.

Chemokines are a group of chemoattractant cytokines released by tissues in the beginning of infection. They are usually produced by different cell types in response to bacterial products and other pathogens. Besides the promotion of immune cells chemotaxis to the site of infection, they regulate a variety of biological processes related to cellular activation, differentiation and survival^61^, such as those found in our study (Supplementary File 1: Table S3). In mice, it has been shown that in the presence of bacterial infection, there is an increase of inflammatory cytokines, which can lead to osteocyte apoptosis and consequently osteonecrosis^62^. In our study, two chemokines (*CCL26* and *CCR5*) were DE in the chicken articular cartilage. The *CCL26* has a bactericidal activity verified against pathogens *Streptococcus pneumoniae, Staphylococcus aureus, Nontypeable Haemophilus influenzae,* and *Pseudomonas aeruginosa*^63^, while *CCR5* has already been identified as biomarker for osteonecrosis of the femoral head in human plasma^64^.

Our results showed several biological processes and genes related to immune response, indicating that the overexpression of these genes is activating the immune system to fight against the progression of FHS, evidencing the presence of an inflammatory process, even at the early stages of FHS.

### Bone-related bioprocesses

The results of the NetworkAnalyst platform indicates associations between DE genes and Wnt signaling pathway, GnRH signaling pathway, Adrenergic signaling in cardiomyocytes*,* VEGF signaling pathway, Bacterial infection, Focal adhesion, Gap junction, Toll-like receptor signaling pathway*,* AGE-RAGE signaling pathway in diabetic complications and NOD-like receptor signaling pathway (Fig. 2). The Wnt signaling is an ancient and evolutionarily conserved pathway responsible for the regulation of crucial aspects of cell fate determination, cell migration, cell polarity, neural patterning and organogenesis during embryonic development^65^, which are important for bone development. Most of the genes enriched in this BP also appeared in focal adhesion and Toll-like receptors. Some of these processes have already been described by Peixoto et al.^12^ and could be intrinsically correlated with FHS.

Durairaj et al.^66^ suggested that FHS could be a metabolic problem, related to fat metabolism disorders, facilitating an unbalanced growth in the articular-epiphyseal complex that leads to its separation under shear stress. They observed that the blood parameters such as cholesterol, triglycerides, and low-density lipoproteins were slightly increased in FHS-affected chickens. Despite the physiological differences between humans and chickens, the appearance of the GnRH signaling pathway, AGE-RAGE signaling pathway in diabetic complication and adrenergic signaling in cardiomyocytes, indicates that chickens may have a similar physiology, needing more studies to better elucidate these pathways.

The genes *MAPK12* and *RAC2* were connected to the VEGF signaling pathway. This pathway is crucial to the vascular development stages and processes, like vasculogenesis, angiogenesis and lymphangiogenesis, which are essential for specification, morphogenesis, differentiation, and homeostasis of vessels during development and in the adulthood^67^. The involvement of VEGF signaling pathway in the FHN and BCO in chickens has already been observed^11,12,20,24^ and could affect the cells regeneration and maintenance^68^. Furthermore, the *MAPK12* gene is located in a QTL for bone mineral density in humans^69^, while the activity of *RAC2* gene has been observed in the osteocalastogenesis^70,71^, involved in the development of tibial dyschondroplasia in chickens^72^ and osteoarthritis in humans^73^.

Moreover, the DE genes identified in the current study were investigated for associations with locomotor problems using the human curated information of DisGeNET database from the NetworkAnalyst 3.0 (Fig. 3). This analysis showed that the *ADA, ANK1, JUN, ACTA1, TNNT3* and *ACTA1* genes were also related to human locomotor problems, evidencing a similar pattern in chickens and humans. Although the knowledge of the chicken transcriptomic profile is increasing, the functional annotation of its genome remains incomplete. In this way, human databases are still needed to infer pathway information in the chicken^74^. Therefore, more studies are needed to better understand the role of these genes in the development of locomotor problems in chickens.

### Response to biotic stimulus, gas transport, cell activation and cytolysis

Biomechanical continuous local stress and impaired blood flow to the epiphyseal-physical cartilage are some of the factors that favor the pathogenesis of osteochondrosis, reported in several animal species^75–80^. The FHS has been associated with the growing phase and a large number of DE genes in this study were involved in BP response to biotic stimulus and regulation of multi-organism processes, which are relevant to the animal locomotor system development. The DE genes associated to FHS in these BP are *ADA, AvBD1, AvBD2, C7, CCND3, CSF3R, EDN2, EPB42, GGT1, JUN, LECT2, LYG2, PTPRC, RAC2, RHAG, RSFR, S100A9, SELP, SERPINB10, STOM.*

One of the main BP enriched in the current study was gas transport and cell migration (Fig. 1). The *RHAG* is one of the genes of the Rh gene family^81^. This gene is usually expressed in tissues that produce blood cells, but it is also expressed in heart cells and those related to the gas transferring system from the lungs to organelles within cells^82,83^. The elevated expression of genes related to the gas transfer system can indicate a more pronounced O_2_ reduction or CO_2_ enhancement. The imbalance between the O_2_ supply and CO_2_ removal of the gas transferring elements has already been associated to hypoxia or hypercapnia, which could lead to the damage of the heart cells metabolism^84,85^. The upregulation of *RHAG* can be related to the proximal FHS development, since in consequence of the pressure in the bone structure, caused by inflammation, the blood supply is reduced, causing hypoxia due to the lack of oxygen. *RHAG* upregulation can also be related to a consequence of FHS since its upregulation leads to increased oxygenation of the affected tissue. This gene was also enriched in several others BP, such as those related to immune response.

The apoptosis is probably involved in the FHS in broilers, since it is a physiological mechanism crucial in the development and tissue homeostasis. In our study, this BP was not enriched in the DAVID database, but some genes associated with apoptosis were DE (*ADA, JUN, IFI6*). The gene *IFI6,* also known *as ISG12*, has an important role in apoptosis regulation^20,86^. In humans, this gene encodes a hydrophobic protein that acts in intracellular signaling^87,88^, but in birds, it does not have its function fully established. Furthermore, the ISGs family is known to generate cellular and physiological diversity and it is associated with antiviral, anti-tumor and immunomodulatory activity mechanisms^89^. In our results, the gene *IFI6* was DE and co-located with the gene *STEAP4*. These genes are expressed at the same site (cell or tissue), and their functions are related to regulation of cellular metabolism during osteoblast differentiation and regulation of apoptosis^90^. The upregulation of the *IFI6* can be related to a causal factor, stimulating an excessive apoptosis at the articular cartilage, turning the animal more susceptible to FHS.

### Extracellular matrix

The carbohydrate derivate catabolism was one of the superclusters observed, which contained the carbohydrate derivate catabolism and glycosaminoglycan catabolic BP (Fig. 1, Supplementary File 1: Table S3). The extracellular matrix (ECM) is a structurally stable component that is located under the epithelium and surrounds connective tissue cells^91^. Due to its structure, ECM is responsible for providing support and resistance to tissues and organs throughout the body, and acts in biochemical processes related to tissue morphogenesis, differentiation and homeostasis^92^. In addition, in the ECM there are molecules, like glycosaminoglycan, responsible for cell modulation, such as adhesion, migration, proliferation, differentiation and cell survival of the tissue^93^.

The glycosaminoglycans are fundamental components fulfilling various ECM biological functions. They are highly polar and can also contribute to permeability properties, connective tissue structure and as a guide to enzymes and growth factors in both the matrix and cell surface^94^. The DE genes enriched in the glycosaminoglycan and aminoglycan BP were upregulated in FHS-affected broilers (*ADA, SERPINB10, AvBD1, STOM, JUN, RHAG, KEL, TF, EDN2, EPB42*) (Supplementary File 1: Table S3). Here, important upregulated genes are *ADA, RHAG* and *JUN,* which participate in the glycosaminoglycan (GAGs) and aminoglycan metabolic processes involved in the ECM metabolism. The *RHAG, ADA* and *AvBD1* differential expression pattern between healthy and FHS-affected group were also confirmed by qPCR (Table 3).

Genes grouped in these previous BP were upregulated in the FHS-affected broilers, indicating that the body tries to fix the damage through remodeling. Altogether, the results indicate that the upregulation of the genes could be a consequence of the damage by the FHS, where the upregulation of those genes is an attempt to diminish the injury, since glycosaminoglycans mediate various receptor-ligand interactions on the cell surface and, as a result, play an important role in development, as well as in lesion repair^94^.

In this study, response to biotic stimulus, immune response and cell activation processes were BP highly represented. FHS may cause important physiological implications to the broiler’s development, which leads to more severe disorders.

There are some studies conducted with chicken bone tissue evaluating locomotor problems, but just one has recently been performed with cartilage tissue^95^. Both tissues are important to the development of those problems. Therefore, the knowledge of the relation between bone and cartilage tissues with these disorders is essential to provide alternative strategies to counteract these complex production problems. The identification of young broilers with vulnerable femoral joint can help genetic selection to reduce this anomaly. The BCO pathology does not show clinical signs at early stages, only at late stages or after necropsy when the diagnostic is possible^14^. The use of infrared thermography (IRT) was suggested as a technique to detect lesions attributed to BCO^96^. The IRT consists of a noninvasive technique that measures infrared radiation from an object and can be a useful tool to evaluate clinical health. Although there are options to confirm the diagnosis of this condition, there are still limitations. The functional analyses of the DE genes help to elucidate their involvement in the development of FHS. These results contribute to a better understanding of the FHN in chickens and possibly other femur disorders in humans.

In summary, the first transcriptome of the femoral articular cartilage was generated, and biological processes and genes involved with femur head separation in rapid growth chickens were identified. Some genes such as *AvBD1, AvBD2, ANK1, RHAG, ADA* and *EPX* were firstly associated to FHS in broilers, indicating that the disruption in the articular cartilage could favor the development of this condition. These results might help the development of strategies to reduce the manifestation of this disorder in poultry, improving welfare and reducing economic losses.

## Material and methods

### Ethics statement

All of the experimental procedures were conducted in conformity with the guidelines of the Ethics Committee for Animal Use (CEUA) from the Embrapa Swine and Poultry National Research Center, with approval protocol number 012/2012, in agreement with the rules of the National Council of Animal Experimentation Control (CONCEA) to ensure compliance with international guidelines for animal welfare.

### Animals and sample collection

A total of 29 Cobb500 commercial male broilers from a poultry farm, located in Concórdia/SC, Brazil, was used in this study. Broilers were housed according to the standard practices, raised with free access to both feed and water. To reduce environmental effects, the broilers used in this study were sampled from the same flock, in a darkhouse system managed by a high standard producer. At the farm, the animals were selected based on the absence or presence of lameness and split into two groups: 14 normal and 15 chickens showing lameness as described by Peixoto et al.^12^. At the necropsy, the animals were evaluated for the presence or absence of FHS according to Wideman and Prisby^7^ and Paludo et al.^11^. Broilers showing separation between femoral GP an AC were included in the FHS-affected group and broilers with good adhesion of the AC and GP were considered normal and were included in the control group, as in Peixoto et al.^12^. From those, eight samples of AC, four normal (average weight 2,401 g ± 31.19) and four FHS-affected (average weight 2,406 ± 148.45 g) were randomly chosen for RNA-Seq, collected in liquid nitrogen and stored at − 80 °C. None of the chosen broilers were visually affected by BCO.

### Total RNA extraction, library preparation and sequencing

For total RNA extraction, eight samples of femoral articular cartilage (4 from each group) were homogenized in liquid nitrogen and 100 mg of tissue was added in 1 mL of TRIzol reagent. Then, 200 μL of chloroform were added, the tubes were homogenized for 15 s and incubated at room temperature for five minutes, centrifuged at 16,000 ×* g* at 4 °C for 15 min. The aqueous phase was separated into a new microtube, mixed with 70% ethanol. This solution was added to a Qiagen RNeasy silica column (Qiagen, Hilden, NRW, Germany), and the RNA extraction followed the standard protocol of Qiagen RNeasy kit (Qiagen, Hilden, NRW, Germany), according to the manufacturer´s instructions. Total RNA was quantified using the BioDrop spectrophotometer (Biodrop, Cambridge, UK) and samples with OD260:OD280 ratio greater than 1.9 were considered pure. The RNA integrity was confirmed in a 1% agarose gel electrophoresed for 90 min with limit of 90 V in 1X TBE buffer and in Agilent 2100 Bioanalyzer (Agilent Technologies, Santa Clara, CA, USA). Samples with RNA integrity number (RIN) greater than 8.0 were used for RNA libraries preparation.

Approximately 2 μg of total RNA was submitted to library preparation using the TruSeq Stranded mRNA Library Prep Kit (Illumina, Inc., San Diego CA, USA), according to the manufacturer's recommendations. The size of the libraries was confirmed in Agilent 2100 Bioanalyzer (Agilent Technologies, Santa Clara, CA, USA). Libraries were quantified with qPCR using primers with Illumina adapters and then sequenced in an Illumina HiSeq2500 (Illumina Inc., San Diego, CA, USA) at the Center for Functional Genomics at ESALQ, University of São Paulo, Piracicaba—SP, Brazil, using HiSeq SBS Kit (Illumina Inc., San Diego, CA, USA) following a paired-end (2 × 100 bp) protocol. All samples were sequenced in the same lane.

### Quality control, mapping and differential expression analyses

The reads quality control (QC) was performed with Trimmomatic v. 0.38^97^ to remove short reads (< 70 bp), low-quality reads (QPhred < 20) and adapter sequences. Mapping was performed with STAR 2.7^98^ using the chicken reference genome (*Gallus gallus*, assembly GRCg6a) available at the Ensembl 95 database (www.ensembl.org). The reads were counted in exon regions using HTSeq v. 0.11.2^99^ and the EdgeR^100^ implemented in R language^101^ was used to identify DE genes between the normal and affected groups. Genes with false discovery rate (FDR) < 0.05 were considered DE, after correcting for the Benjamini-Hochberg (BH) multiple-tests^102^. Genes were considered upregulated and downregulated according to the positives and negatives log2 fold-change (Log2FC), respectively, in the affected compared to the normal broilers. The multidimensional scaling (MDS) plot was created using the normalized read counts for each sample using the plotMDS function in edgeR^100^. Based on DE genes, a heatmap was generated to check the consistence between samples using the heatmap.2 function from gplots in R^101^. The FASTQ files sequenced in this study were deposited in the SRA database, with BioProject number PRJNA350521.

### qPCR validation

To confirm the results obtained in the RNA-Seq analysis, a quantitative PCR analysis (qPCR) was performed using the same eight AC samples from the normal and FHS-affected animals. Total RNA was extracted as described above. The cDNA synthesis was performed according to the recommendations of the SuperScript III, First-Strand Synthesis Supermix protocol (Invitrogen, Carlsbad, CA, USA). For validation, 11 DE genes in the RNA-Seq analysis were chosen based on FDR, Log2FC and their function as candidates to be involved with FHN: avian beta-defensin 1 (*AvBD1*), avian beta-defensin 2 (*AvBD2*), fibrillin 2 (*FBN2*), ankyrin 1 (*ANK-1*), phosphoserine phosphatase (*PSPH*), eosinophil peroxidase (*EPX*), adenosine deaminase (*ADA)*, collagen type XIII alpha 1 chain (*COL13A1*), Rh associated glycoprotein (*RHAG*) and S100 calcium-binding protein A9 (*S100A9*). Primers were designed using the NCBI Primer-BLAST tool^103^ (Table 4) and the quality was evaluated and confirmed with the Netprimer program (http://www.premierbiosoft.com/NetPrimer). For the relative quantification analyses, reactions were prepared using 1X GoTaq qPCR Master Mix (Promega, Madison, WI, USA), with BRYT Green Dye and CRX as reference dye, 0.13 μM of forward and reverse primers, 2 μL cDNA at 1:10 dilution and ultrapure water (Nuclease Free Water, Qiagen) to complete a 15 μL reaction. The reactions were performed in duplicate and submitted to the QuantStudio 6 Real-Time PCR equipment (Applied Biosystems, Foster City, CA, USA) with an initial cycling of 95 °C for 3 min, followed by 40 cycles of 95 °C for 15 s and 60 °C for one minute, with melting curve of 95 °C for 15 s, 60 °C for 1 min and 95 °C for 15 s.

**Table 4:** Primers used for the qPCR analysis of the target candidate genes for FHS in the femur articular cartilage of broilers.

Gene	Ensembl ID	Primer sequences (5'-3')
***AvBD1***	ENSGALG00000022815	F: CAGGATCCTCCCAGGCTCTA
avian beta-defensin 1		R: GATGAGAGTGAGGGAAGGGC
***AvBD2***	ENSGALG00000016669	F: TTCTCCAGGGTTGTCTTCGC
avian beta-defensin 2		R: TGCATTCCAAGGCCATTTGC
***FBN2***	ENSGALG00000014686	F: TGCATCGATAGCCTGAAGGG
fibrillin 2		R: CTAATTCACACCGCTCACATGG
***ANK1***	ENSG00000029534	F: CCACCATCCCACCATTCAGT
Ankyrin 1		R: ACGGTCACAAACTCCAGCAT
***PSPH***	ENSGALG00000002397	F:CAGGAATACGGGAGCTGGTG
phosphoserine phosphatase		R: CCCAGAGACCAGGAAGACCT
***EPX***	ENSGALG00000043254	F:AAAGGAGGTGGCATTGACCC
eosinophil peroxidase		R: GCCACGCTGCATGTTAAGAG
***ADA***	ENSGALG00000004170	F:TTCGGCAAGAAAAGAGGGGT
adenosine deaminase		R: GTGTTTGGTAGCTGACGTGC
***COL13A1***	ENSGALG00000004286	F:CCAAGCAAGGACTAGACACTCA
collagen type XIII alpha 1 chain		R: ACCCTTCATGCCATGTCTTCC
***CSF3R***	ENSGALG00000002112	F: TCATCCGGGACAGCATTGAG
colony stimulating factor 3 receptor		R: TGTAGAGGGGGTACACCGAG
***RHAG***	ENSGALG00000016684	F:TCTGGAGATCACGGCCTTTG
Rh associated glycoprotein		R:GCTCCAATATCTGTGGCCTGA
***S100A9***	ENSGALG00000024272	F:GGGGACAAAGACACCCTGAC
S100 calcium binding protein A9		R:TTCACGTGCTTCAGGTAGTTGG

The Ct means of each sample were obtained to perform the relative expression ratio analysis^104^. For the data normalization, the geometric mean of the Ct values from the reference genes *RPL5* (*Ribosomal Protein L5*) and *RPLP1* (*Ribosomal Protein Lateral Stalk Subunit P1*) were used. These genes were chosen based on their stability evaluation in chicken femoral articular cartilage^105^. The Relative Expression Software Tool (REST)^104^ was used to perform the relative quantification and the statistical test, using the non-parametric Pair Wise Fixed Reallocation Randomization Test^106^. Genes with p-values ≤ 0.05 were considered DE.

### Functional annotation

To investigate the role of DE genes in known metabolic pathways, the list of all DE genes was analyzed with the Functional Annotation Clustering (FAC) implemented in the DAVID database (http://david.abcc.ncifcrf.gov/)^107,108^, considering a p-value of < 0.10 as significant. Subsequently, the biological processes were clustered in the REVIGO^109^. An enrichment analysis was also performed using the chicken genome in the NetworkAnalyst^110^, where new biological functions were obtained with the protein–protein interactions (PPI). Moreover, a gene-disease association network was created with the DE genes using human curated information of DisGeNET database available in the NetworkAnalyst^110^, considering a *p*-value of < 0.05 as significant.

## Supplementary Information

Supplementary Information 1.

Supplementary Information 2.

## Data Availability

The datasets generated during and/or analyzed during the current study are available from the corresponding author on reasonable request. The fastq files used in this study were submitted to SRA database and will be available as BioProject number PRJNA350521.
