# Supplementary material for: Differentially expressed genes in the femur cartilage transcriptome clarify the understanding of femoral head separation in chickens
Source: Sci Rep. 2021 Sep 9;11:17965. doi: 10.1038/s41598-021-97306-3 (PMC8429632; doi:10.1038/s41598-021-97306-3)
Supplement: Supplementary file 2 — Supplementary Information 2. [file 41598_2021_97306_MOESM2_ESM.docx]

**Differentially expressed genes in the femur cartilage transcriptome clarify the understanding of femoral head separation in chickens**

**Ludmila Mudri Hul, Adriana Mércia Guaratini Ibelli, Igor Ricardo Savoldi, Débora Ester Petry Marcelino, Lana Teixeira Fernandes, Jane de Oliveira Peixoto, Maurício Egídio Cantão, Roberto Hiroshi Higa, Poliana Fernanda Giachetto, Luiz Lehmann Coutinho, Mônica Corrêa Ledur**

# Supplementary file 2

#
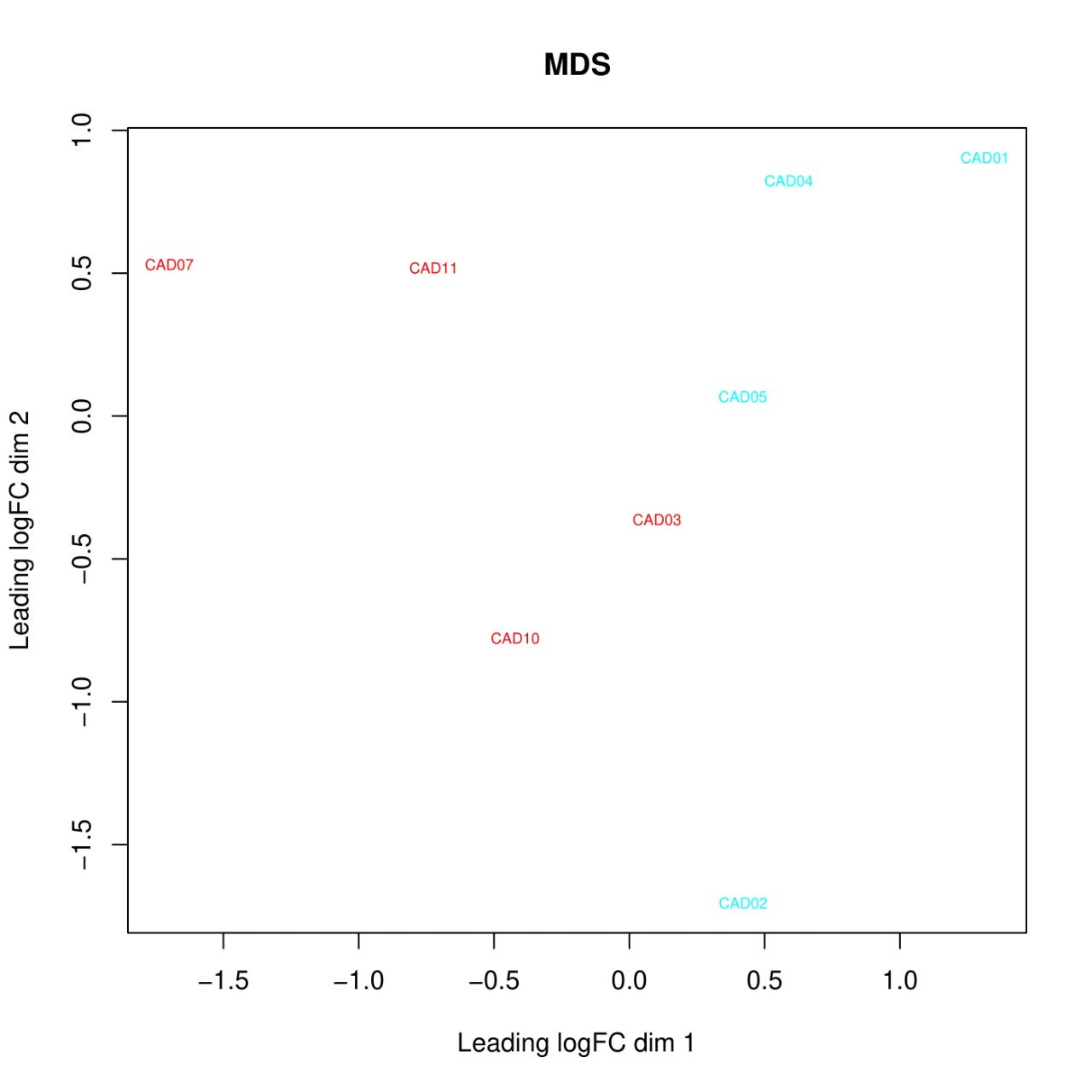


**Fig S1.** Multidimensional scale (MDS) plot showing the separation between samples from normal (red) and FHS-affected broilers (blue).


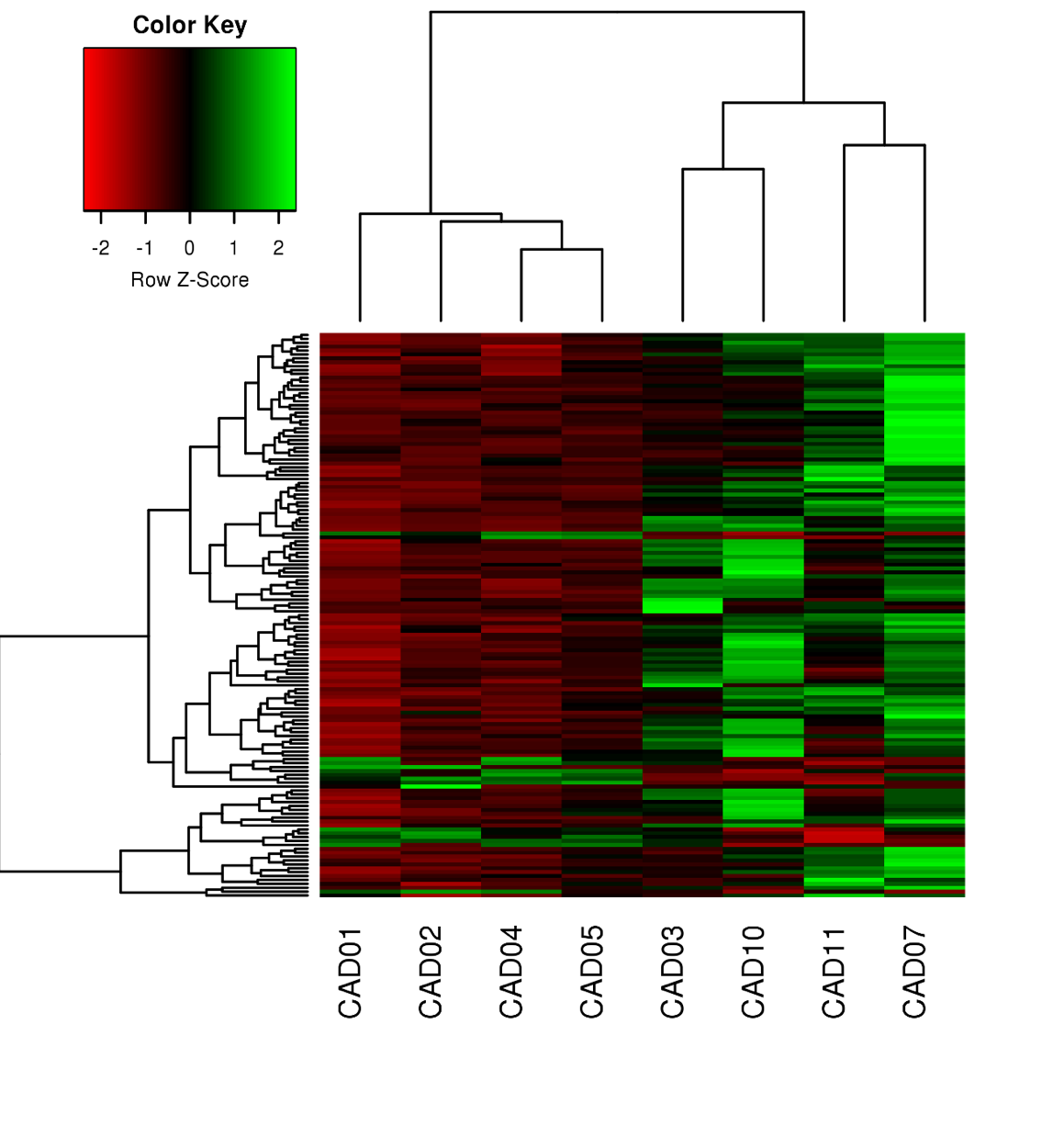


**Fig S2.** Heatmap with 107 differentially expressed genes between samples of the femur head cartilage tissue from normal (01, 02, 04, 05) and FHS-affected broilers (03, 07, 10, 11). Each gene expression is shown in the lines and the samples in columns, resulting in a hierarchical group of genes and samples. In red are the downregulated and in green are the upregulated genes in the affected animals.
